# Combined ^18^F-AlF-NOTA-FAPI-04 and ^18^F-Fluorodeoxyglucose PET/CT in patients with eosinophilic gastroenteritis: a case report and literature review

**DOI:** 10.3389/fmed.2025.1602306

**Published:** 2025-08-29

**Authors:** Bo Chen, Tong Wu, Jinghui Xie

**Affiliations:** ^1^Department of Nuclear Medicine, The First Affiliated Hospital of Dalian Medical University, Dalian, China; ^2^Department of Oncology, The First Affiliated Hospital of Dalian Medical University, Dalian, China

**Keywords:** FDG, FAPI, PET/CT, eosinophilic gastroenteritis, peritoneal

## Abstract

Eosinophilic gastroenteritis (EGE) is a rare chronic inflammatory disorder characterized by eosinophilic infiltration of the gastrointestinal tract. We report the case of a 42-year-old previously healthy man who presented with gradually worsening abdominal pain and bloating for approximately 1 month. Initial laboratory tests showed elevated eosinophil counts, increased immunoglobulin E levels, and raised C-reactive protein. Enhanced CT revealed diffuse edema of the gastric wall, thickening of the gastric and duodenal walls, enlargement of the abdominal lymph nodes, and thickening of the peritoneum, which was suspected to be caused by malignant tumors. However, gastroscopic pathological examination and multiple ascites examinations showed no obvious malignant cells. To investigate the underlying cause, the combined ^18^F-AlF-NOTA-FAPI-04 (^18^F-FAPI) positron emission tomography/computed tomography (PET/CT) and ^18^F-Fluorodeoxyglucose (^18^F-FDG) PET/CT were performed. The pattern of increased radionuclide uptake in these mentioned lesions differs from that of malignant neoplasms. Then, EGE was confirmed by diagnostic peritoneal biopsy. This case highlights that PET/CT imaging combined with ^18^F-FAPI and ^18^F-FDG demonstrates potential utility in diagnosing EGE, particularly in distinguishing inflammatory processes from malignancies.

## 1 Introduction

Eosinophilic gastroenteritis (EGE) is a rare gastrointestinal disorder, most commonly affecting the stomach and duodenum (1). The prevalence of EGE is approximately 1.7/100,000, which is more common in young and middle-aged people aged 30–50 years (2, 3). The clinical symptoms of EGE vary according to the site and depth of involvement (4). Mucosal EGE is the most common type, characterized by nausea, vomiting, abdominal pain, diarrhea, and decreased weight. The muscular type presents with thickening of the gastrointestinal wall, rigidity of the intestinal lumen, and potential obstruction. The serosal type is characterized by large amounts of ascites and abdominal distension, accompanied by marked eosinophilic infiltration (5, 6).

Studies have shown that up to 60–70% of patients exhibit peripheral eosinophilia and elevated immunoglobulin E levels (6), but both lack specificity. In one-third of EGE patients, barium contrast studies appear normal, with mucosal fold thickening being the most notable imaging feature (7). CT and MRI findings in EGE correlate with the location and depth of eosinophil infiltration in the gastrointestinal wall, such as the characteristic “target sign” and “tram-track sign” in small intestinal EGE, which aid in diagnosis (1, 8). Endoscopic findings in EGE patients commonly include mucosal congestion and edema, followed by erosion (5). However, these imaging and endoscopic features lack specificity. Endoscopic tissue biopsy is a crucial diagnostic tool for EGE (9). Definitive diagnosis requires a histopathological demonstration of >20 eosinophils/HPF. However, the heterogeneity of eosinophilic infiltration at different sites poses a challenge, particularly for lesions confined to the muscular or subserosal layers, where the mucosal layer may appear normal (10, 11), which brings great distress to the clinical diagnosis of EGE. While multiple biopsies (including duodenum) of endoscopy were recommended (10, 12), missed or misdiagnosed cases remain unavoidable, as demonstrated in this case.

To date, ^18^F-fluorodeoxyglucose (^18^F-FDG) positron emission tomography/computed tomography (PET/CT) imaging of EGE has only been reported in a few cases (13–16), while ^18^F-AlF-NOTA-FAPI-04 (^18^F-FAPI) PET/CT imaging remains poorly understood (17). In this study, we present a case of EGE evaluated with both ^18^F-FAPI and ^18^F-FDG PET/CT and review relevant literature to enhance the understanding of this condition. We emphasize the role of dual-tracer PET/CT in the diagnostic workup of EGE, particularly in excluding gastrointestinal malignancies.

## 2 Case presentation

**Figure 1:**
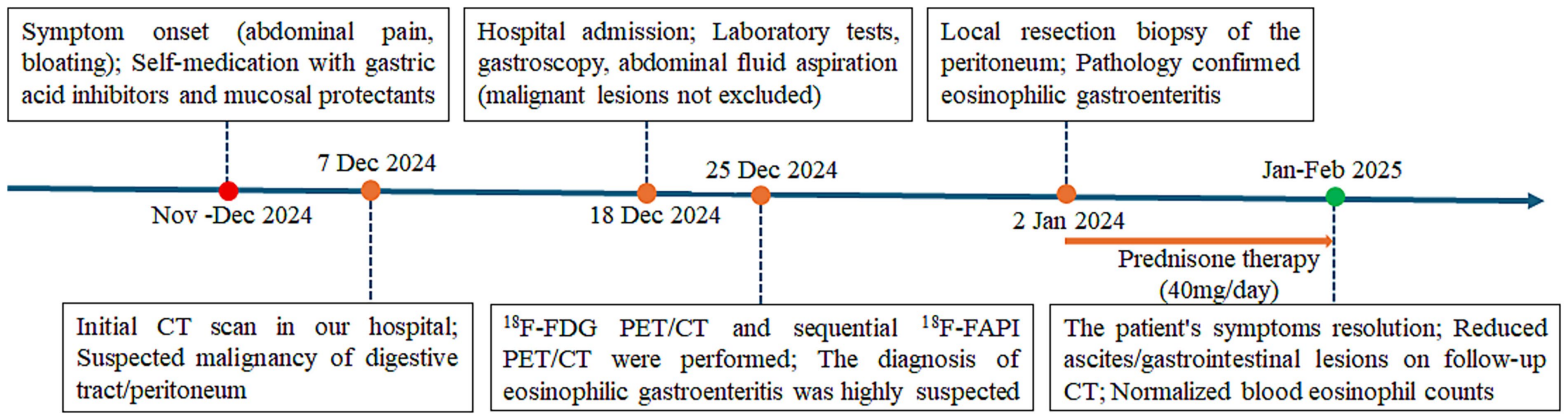
Timeline of the clinical course and treatment of the patient with eosinophilic gastroenteritis.

A timeline of the onset, diagnosis, and therapy is shown in Figure 1. We present the case of a 42-year-old man who was admitted to our hospital on 7 December 2024, presenting with a 1-month history of abdominal distension, epigastric discomfort, and bilateral hypochondrial pain, accompanied by anorexia, mild fatigue, and 5 kg weight loss. Symptoms persisted despite self-administered gastric acid suppression and mucosal protectants. Physical examination showed abdominal distension with shifting dullness but no tenderness or masses. Laboratory tests revealed elevated blood eosinophil count (0.67 × 10^9^/L, reference range 0.02–0.52 × 10^9^/L), tumor marker CA125 (111.47 U/mL, reference range 0–35 U/mL), erythrocyte sedimentation rate (31.00 mm/h, reference range 0–15 mm/h), C-reactive protein (275.8 mg/L, reference range 0–5.0 mg/L), and immunoglobulin E (273 IU/mL, reference range 0–100 IU/mL). His tuberculosis screening, rheumatoid factor, amylase, and lipase were normal. Additionally, the patient had no history of past medical issues.

**Figure 2:**
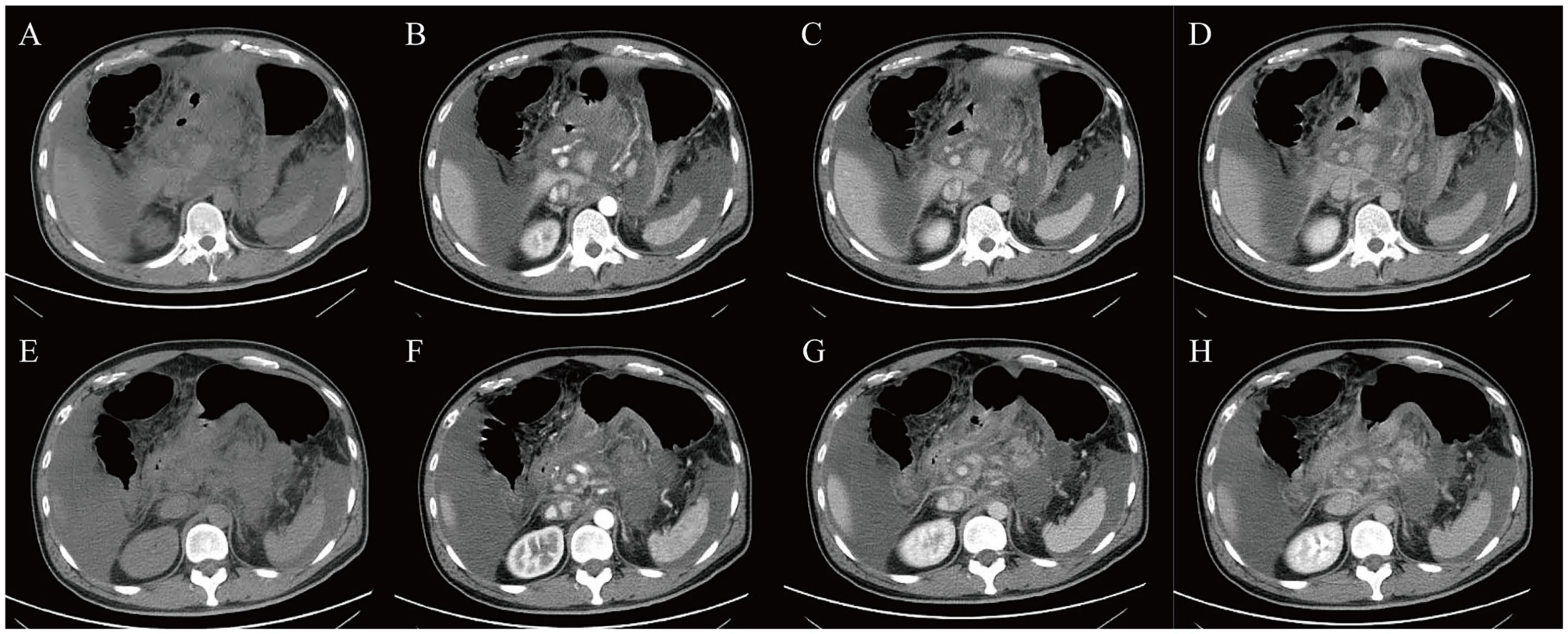
Contrast-enhanced CT scans. **(A,E)** plain CT, **(B,F)** enhanced CT arterial phase, **(C,G)** enhanced CT venous phase, **(D,H)** enhanced CT delayed phase.

A three-phase enhanced scan of the entire abdomen was performed by Discovery HD CT (GE Healthcare, Chicago, USA). The images showed thickening of the gastric antrum and body walls, with the maximum thickness part reaching 2.3 cm. The CT value was approximately 22 Hu, and moderate heterogeneous enhancement was observed, with three-phase CT values of 41 Hu, 77 Hu, and 47 Hu, respectively. Contours were poorly defined, with blurred surrounding fat spaces and enlarged lymph nodes along the lesser curvature of the stomach. The proximal duodenal wall appeared irregular, and the pancreatic contour was coarse, but the density and enhancement were homogeneous. The amount of fluid density was observed in the abdominal cavity (Figure 2).

**Figure 3:**
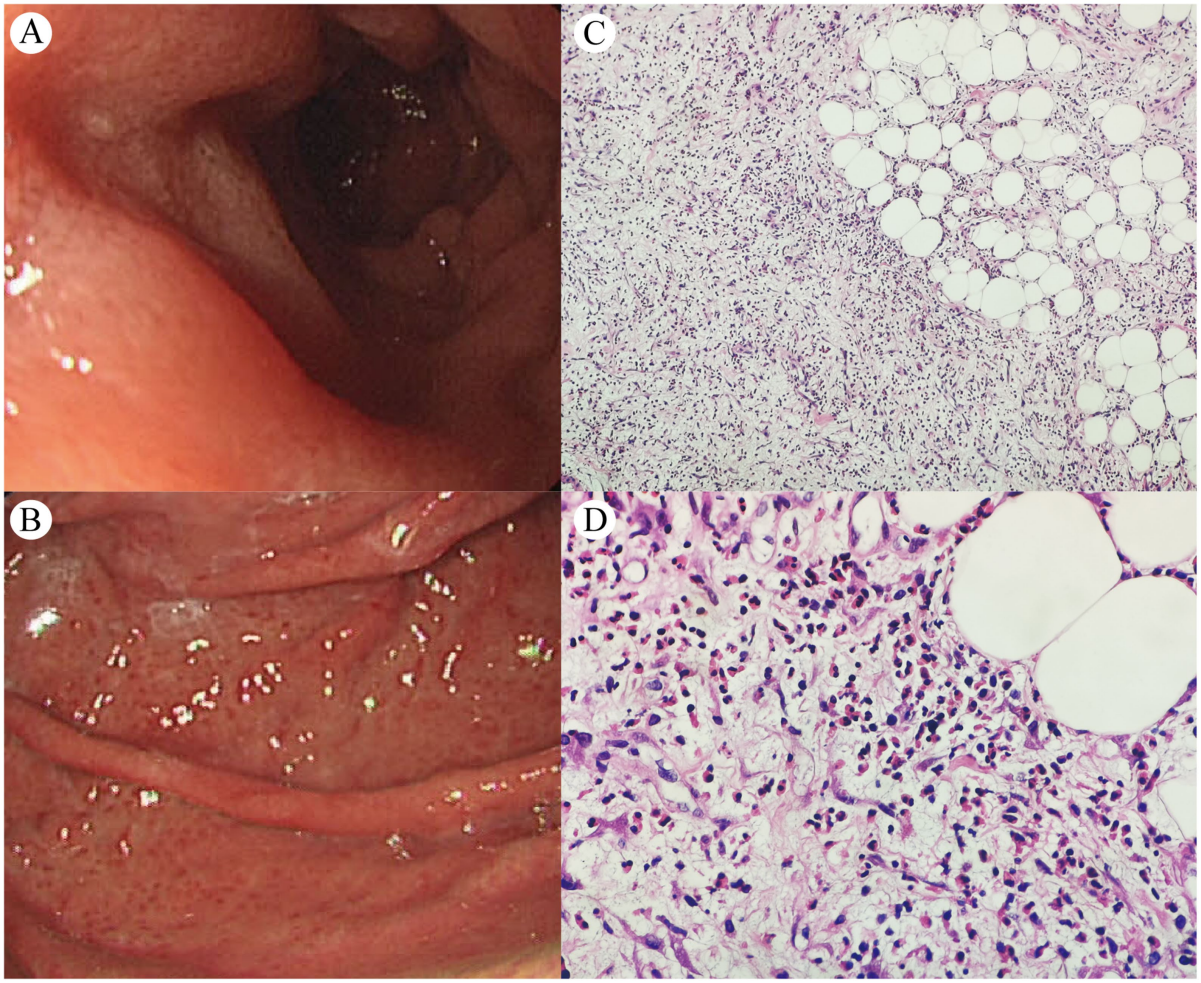
Upper gastrointestinal endoscopy **(A,B)** and peritoneal histopathology of the biopsy specimen [**C** (×100) and **D** (×400)].

Upper gastrointestinal endoscopy (Figures 3A,B) revealed extensive congestive blood spots on the gastric fundus and body wall, with attached white exudates, stiff and coarse folds, poor extension after inflation, and rough and edematous mucosa in the gastric sinus. The lumen of the descending part of the duodenum was slightly narrowed, but no mucosal abnormality was observed. Multi-point mucosal sampling and biopsy were performed; pathology revealed chronic active inflammation with intestinal metaplasia. Multiple aspirations of ascites were performed, which appeared yellowish and turbid, with a specific gravity of 1.032, protein positivity (+), total cell counts of 2,200/μL, nucleated cell counts of 1,241/μL, eosinophil proportion in ascites of 14–20%, protein quantification of 48.7 g/L, adenosine deaminase (ADA) of 10.5 U/L, and lactate dehydrogenase (LDH) 197 U/L. Cytopathological examination of ascites showed no atypical cells.

**Figure 4:**
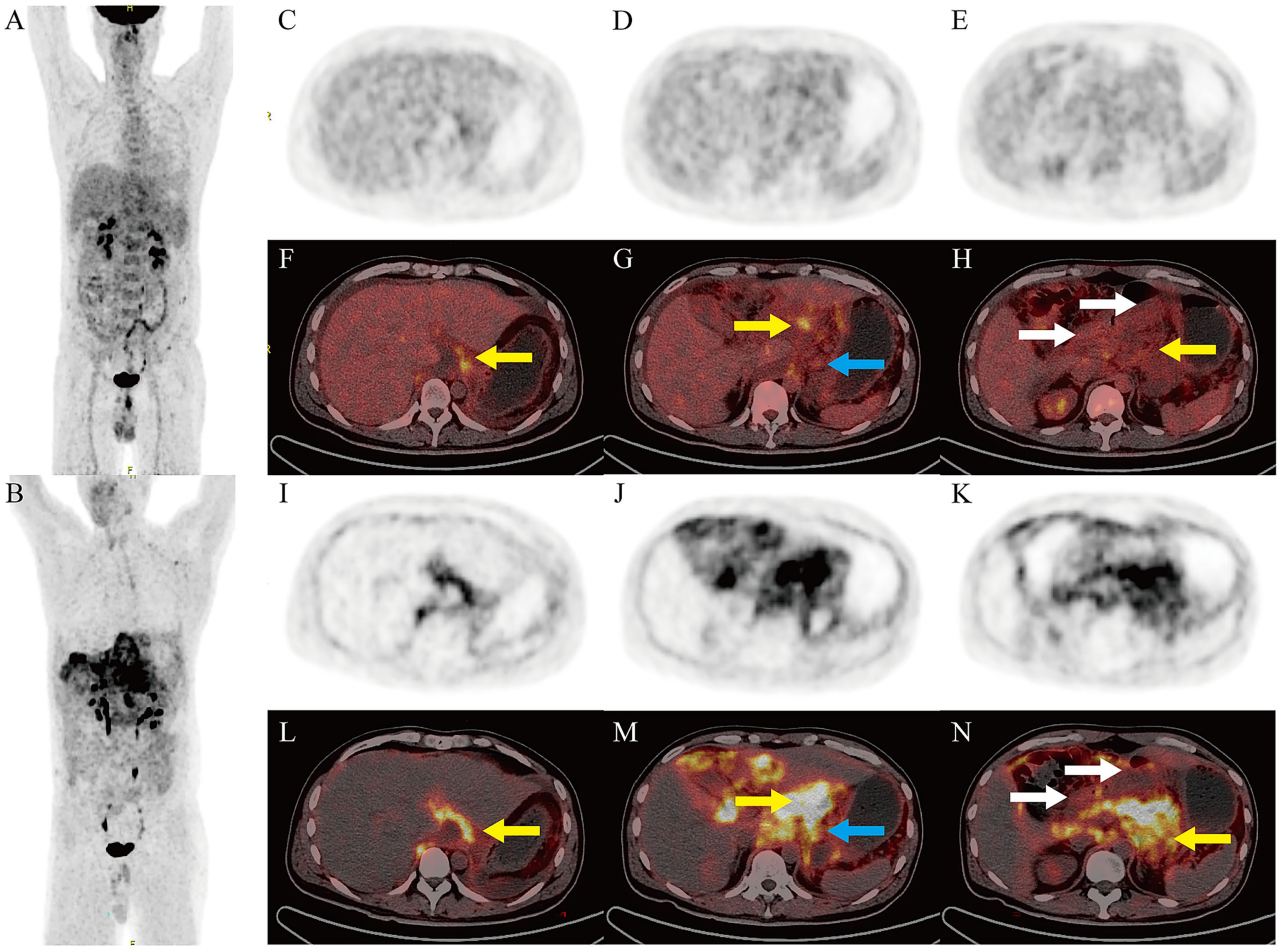
PET/CT Imaging. Maximum intensity projection of ^18^F-FDG **(A)** and ^18^F-FAPI **(B)** PET. Axial ^18^F-FDG PET **(C–E)** and PET/CT fusion scans **(F–H)**, axial ^18^F-FAPI PET **(I–K)**, and PET/CT fusion scans **(L–N)**.

Subsequently, a Biograph True-Point Row 64 PET/CT (Siemens Medical Solutions, Erlangen, Germany) was performed for differentiating diagnosis (Figure 4), ^18^F was produced using a Siemens Eclipse cyclotron, ^18^F-FDG and ^18^F-FAPI radiochemical synthesis was performed using the All in One® (Tarsias, Ans, Belgium) and Explora FDG4 (Siemens Medical Solutions, Erlangen, Germany) Synthesis Module. The radiochemical purities were both greater than 95%. Yellow arrows: Diffuse heterogeneous peritoneal thickening (hepatogastric ligament, lesser omental bursa, greater omentum, pancreatic anterior, left renal fascia) with mildly increased ^18^F-FDG uptake (SUVmax 3.9–5.5; Figures 4F–H) and markedly elevated ^18^F-FAPI uptake (SUVmax 6.7–12.9; Figures 4L–N), the latter revealing a larger lesion extent. White arrows: Thickening and edema of the gastric lesser curvature, antrum, duodenal bulb, and descending intestinal wall, with luminal narrowing and rough serosal surfaces. PET showed mild ^18^F-FDG (SUVmax 2.8; Figure 4H) and ^18^F-FAPI uptake (SUVmax 5.7; Figure 4N). Blue arrows: Slightly enlarged lesser-curvature lymph nodes (1.7 cm) with mild ^18^F-FDG (SUVmax 3.6; Figure 4G) and ^18^F-FAPI uptake (SUVmax 8.7; Figure 4M), accompanied by ascites.

Taken together, the patient’s ascites test indicated low cell count but elevated specific gravity and protein levels, suggesting exudative ascites; the patient had no underlying liver, kidney, or heart disease. Tuberculosis, as a granulomatous lesion, often shows significant uptake of ^18^F-FDG on PET scans (18), while ^18^F-FAPI only shows slightly higher metabolism around the granuloma, and the patient’s tuberculosis test was negative, thereby ruling out these causes (19). In this patient, the denial distribution of peritoneal thickening lesions was more significant at the diaphragmatic dome and organ ligament sites, which was different from the common implantation metastasis pattern of carcinomatous peritonitis. Notably, ^18^F-FAPI is a positive imaging modality presenting advantages for signet ring cell carcinoma (SRCC), which did not show significantly high uptake in gastrointestinal lesions in this patient (20). Thus, we also questioned carcinomatous peritonitis, and in combination with other clinical test results, non-specific gastrointestinal inflammation, such as EGE, was considered.

Finally, diagnostic laparoscopy with omental biopsy (Figures 3C,D) revealed mesothelial hyperplasia, fibrous tissue proliferation with fibrinoid necrosis, and marked eosinophilic infiltration (90–120/HPF) with focal fat necrosis. Following diagnosis, the patient initiated oral prednisone therapy at 40 mg/day, with no significant adverse effects observed. After over 2 weeks of treatment, abdominal discomfort symptoms were alleviated, with a follow-up complete blood count showing normalization of eosinophil levels. A follow-up CT scan performed at another hospital on February 2025 showed resolution of ascites and reduced gastric antral/peritoneal thickening; continued follow-up and treatment were recommended. The patient declined repeat gastroscopy and PET/CT surveillance due to procedural anxiety and financial constraints, remaining under clinical monitoring.

## 3 Discussion

EGE is a rare condition characterized by eosinophilic infiltration of the gastrointestinal tract and can involve anywhere from the esophagus to the rectum (1). Its clinical manifestations, often non-specific digestive symptoms, are closely related to the location and depth of eosinophil infiltration (2). Due to its non-specific clinical manifestations, EGE is often misdiagnosed or overlooked, particularly when symptoms overlap with other abdominal pathologies. In this case report, we describe for the first time an EGE with extensive peritoneal involvement who underwent combined ^18^F-FDG and ^18^F-FAPI PET/CT imaging, and this case report details the clinical course, diagnostic work-up, and follow-up of patients with EGE in order to deepen the understanding of this disease.

^18^F-FDG PET/CT, as a well-established imaging modality for visualizing glucose metabolism, is widely recognized in clinical practice (21). Previous data suggest that ^18^F-FDG PET, as a non-invasive imaging modality, holds potential for detecting gastrointestinal inflammatory conditions (22). FAP, a type II transmembrane glycoprotein belonging to the dipeptidyl peptidase family, is highly expressed in cancer-associated fibroblasts within the tumor microenvironment. In recent years, FAPIs have emerged as a novel class of radiotracers for PET imaging (23–25). Compared to conventional imaging agents such as ^18^F-FDG, FAPI tracers offer unique advantages in tumors with low glucose metabolism or high stromal content. The combination of the two effectively improves the disease spectrum of PET/CT for tumor diagnosis, such as well-differentiated carcinoma, signet ring cell carcinoma, and soft tissue sarcoma (23–27). However, their uptake is not exclusive to malignant lesions. It is widely known that the false-positive uptake of ^18^F-FDG can be caused by inflammation, infection, or other benign lesions. FAPI PET imaging may produce false-positive results for non-malignant tumors, which are mainly related to inflammation and fibroblasts. Recent studies have revealed that FAP is often expressed in wound healing, tissue remodeling, and chronic inflammation, leading to growing interest in the application of ^18^F-AlF-NOTA-FAPI-04 (^18^F-FAPI) PET in these areas (28–31). These non-specific uptakes present both challenges and opportunities for PET/CT imaging.

Here, we report a case of EGE that mimicked signet ring cell carcinoma with extensive peritoneal metastasis. In this case, the peritoneal lesions exhibited intense ^18^F-FAPI uptake, with more extensive detection than ^18^F-FDG PET. In contrast, gastrointestinal lesions and lymph nodes demonstrated only slightly increased ^18^F-FDG and ^18^F-FAPI uptake, distinct from gastrointestinal signet ring cell carcinoma with peritoneal metastases, underscoring its potential integration into clinical practice. There was heterogeneity in the levels of ^18^F-FDG uptake between the present case and previously reported gastrointestinal EGE (13–16), with SUVmax ranging from 2.8 ~ 7.1, and we speculate that this may be related to the different periods of the disease. The degree of ^18^F-FAPI uptake was similar to that of the only other case (17) of small bowel EGE (SUVmax 5.7 ~ 7.3), while the unique sign in the present case was that the peritoneum was involved and its extent was more extensive on FAPI than FDG, combined with massive ascites and lymph node enlargement. These findings emphasize that combined ^18^F-FAPI and ^18^F-FDG PET/CT can be used as a potential non-invasive method for evaluating eosinophilic gastrointestinal diseases, which opens new avenues for its application in the field of non-oncology.

However, this is merely an exclusionary empirical diagnosis; this study is a case-by-case analysis, which limits its wider applicability. These findings cannot be used as a basis for diagnosing benign lesions, and further research is warranted to elucidate the underlying mechanisms of radionuclide uptake under these conditions and optimize its diagnostic and therapeutic applications.

## 4 Conclusion

The combined application of ^18^F-FAPI PET/CT and ^18^F-FDG PET/CT offers significant diagnostic value for EGE. However, the rarity of EGE limits large-scale studies, necessitating further evidence to validate PET/CT’s role. Biopsy remains the diagnostic gold standard.

## Data Availability

The original contributions presented in the study are included in the article/supplementary material, further inquiries can be directed to the corresponding author.
